# Functional Characterization and Promoter Analysis of a MeJA-Responsive Squalene Synthase Gene *CpSQS* from *Cyclocarya paliurus* (Batal.) Iljinsk

**DOI:** 10.3390/ijms27188432

**Published:** 2026-09-21

**Authors:** Juan Chen, Qinghui Xia, Yulan Dong, Yuhang Pan, Quanwei Lv, Jinbo Huang, Shaopeng Zhang, Xingxing Dong, Xiaoling Chen

**Affiliations:** 1 School of Modern Industry for Selenium Science and Engineering, Wuhan Polytechnic University, Wuhan 430023, China; 13438789895@163.com (J.C.); 15571778723@163.com (Q.X.); 17603983279@163.com (Y.D.); p2117815912@163.com (Y.P.); taifang19820118@163.com (Q.L.); 13975622379@163.com (J.H.); shaopeng@whpu.edu.cn (S.Z.); 2 National R&D Center for Se-Rich Agricultural Products Processing Technology, Wuhan Polytechnic University, Wuhan 430023, China

**Keywords:** *Cyclocarya paliurus*, methyl jasmonate, triterpenoid biosynthesis, promoter analysis, squalene synthase

## Abstract

*Cyclocarya paliurus* leaves are rich in bioactive triterpenoids, yet their regulatory mechanisms, particularly in response to methyl jasmonate (MeJA), remain unclear. This study aimed to characterize the spatial and temporal effects of MeJA on triterpenoid accumulation, identify MeJA-responsive genes involved in triterpenoid biosynthesis, and functionally evaluate *CpSQS* and its promoter regulatory regions. Upper and lower leaves were sampled at multiple time points after MeJA treatment. Six triterpenoid monomers were quantified by HPLC, and transcriptomes were analyzed at 5 and 10 days. MeJA altered triterpenoid accumulation in a time- and leaf-position-dependent manner, with stronger responses in upper leaves. Differentially expressed genes were enriched in terpenoid backbone biosynthesis, sesquiterpenoid and triterpenoid biosynthesis, plant hormone signaling, and MAPK pathways, while *HMGR*, *FPPS*, *SQS*, and *SE* were generally upregulated. *CpSQS* was induced by MeJA and showed a spatial expression pattern associated with triterpenoid accumulation. Heterologous overexpression of *CpSQS* in tobacco calli significantly increased *CpSQS* transcript abundance and total triterpenoid content, supporting its positive role in triterpenoid biosynthesis. Promoter deletion analysis further identified a 188 bp region from −961 to −773 bp that contributed substantially to promoter activity, with its deletion reducing GUS activity by 56%. These findings reveal the spatiotemporal regulation of triterpenoid biosynthesis by MeJA and identify *CpSQS* and its transcriptionally active promoter region as potential targets for further functional studies and metabolic improvement in *C. paliurus*.

## 1. Introduction

*Cyclocarya paliurus* (Batal.) Iljinsk. (Juglandaceae) is a rare monotypic tree species endemic to China, widely distributed in the southern regions. It is commonly known as sweet tea tree [1]. The leaves of *C. paliurus* are rich in various physiologically active medicinal constituents, including triterpenoids, polysaccharides, and flavonoids [2,3,4]. Modern pharmacological research indicates that extracts and derivatives of *C. paliurus* exhibit multiple biological activities, including antitumour, antioxidant, antibacterial, and hypoglycaemic effects [5]. Its leaves accumulate abundant bioactive secondary metabolites, among which triterpenoids are the major component. These triterpenoids confer *C. paliurus* great potential as a resource for functional foods and pharmaceuticals, yet the molecular regulatory mechanisms underlying their biosynthesis remain unclear.

The biosynthesis of plant triterpenoids originates from the cytosolic mevalonate (MVA) pathway [6]. Squalene synthase (SQS) catalyzes the condensation of two molecules of farnesyl pyrophosphate (FPP) to form squalene, acting as a key enzyme in triterpenoid and sterol biosynthetic pathways [7]. Research indicates that SQS enzyme activity directly influences triterpenoid compound yield in plants [8], making it a crucial target for elucidating triterpenoid biosynthetic regulatory mechanisms. To date, *SQS* genes have been successfully cloned and functionally validated in various plants, confirming its core regulatory role in the triterpenoid synthesis network [9,10].

Plant hormones are endogenous signaling molecules that play pivotal roles in regulating growth and development, enhancing stress resistance, and inducing secondary metabolite synthesis [11]. Under unfavorable environmental conditions such as drought, salinity, wounding and pathogen attack, plants do not simply grow but actively reallocate resources toward survival and defense. As a central stress-associated phytohormone network, jasmonates and their cross-talk with abscisic acid, salicylic acid and auxin orchestrate this trade-off between growth and defense [12]. Upon stress perception, jasmonoyl-isoleucine (JA-Ile) accumulation promotes the degradation of JAZ repressors, releasing the master transcription factor MYC2 and thereby reprogramming a large set of defense- and stress-responsive genes [13,14]. This JA-mediated transcriptional activation not only induces antioxidant systems, stomatal closure and damage repair, but also broadly triggers the biosynthesis of defensive specialized metabolites [12]. MeJA, a derivative of jasmonic acid, can activate the transcription of key genes in the triterpenoid biosynthesis pathway [15]. It has been demonstrated that MeJA significantly upregulates *SQS* expression and promotes squalene accumulation in other medicinal plants [16,17]. Previous studies in autotetraploid *C. paliurus* have shown that MeJA treatment promotes triterpenoid accumulation in the leaves and that this accumulation is significantly and positively correlated with *CpSQS* expression [18,19], suggesting a regulatory association among MeJA signaling, *CpSQS* transcription, and triterpenoid biosynthesis. However, the in vivo functional role of *CpSQS* and the transcriptional regulatory mechanism by which MeJA modulates *CpSQS* expression remain poorly defined.

Gene expression is primarily controlled by promoters, which contain cis-acting elements that bind transcription factors [20]. The cloning and functional analysis of promoters are crucial for deciphering gene regulatory networks. For instance, 5′-deletion analysis has been widely applied to identify key functional regions within promoters, including the 5′-untranslated region (5′UTR) that maintains basal promoter activity [21]. Despite these advances, the promoter of *CpSQS* has not been cloned, and the cis-acting elements responsible for its regulation, particularly in response to MeJA, remain unknown.

Based on the positive association between *CpSQS* expression and triterpenoid accumulation observed in previous studies, we hypothesized that MeJA enhances triterpenoid accumulation in *C. paliurus* through coordinated transcriptional regulation of the triterpenoid biosynthetic pathway, with *CpSQS* serving as an important responsive component. We further hypothesized that the *CpSQS* promoter contains cis-regulatory region(s) that contribute substantially to its transcriptional activity.

Therefore, this study aimed to: (1) characterize the temporal and spatial changes in triterpenoid accumulation and transcriptomic responses in *C. paliurus* leaves following MeJA treatment; (2) evaluate the biological role of the MeJA-responsive *CpSQS* gene through heterologous overexpression in tobacco calli; and (3) characterize the cis-acting elements of the *CpSQS* promoter and identify promoter regions contributing to high transcriptional activity using 5′-deletion constructs, transient expression, and GUS assays. This study provides new insights into the molecular regulation of MeJA-induced triterpenoid biosynthesis and identifies potential genetic and cis-regulatory targets for the metabolic improvement of *C. paliurus*.

## 2. Results

### 2.1. Quality Analysis of Six Triterpenoid Monomers

**Figure 1:**
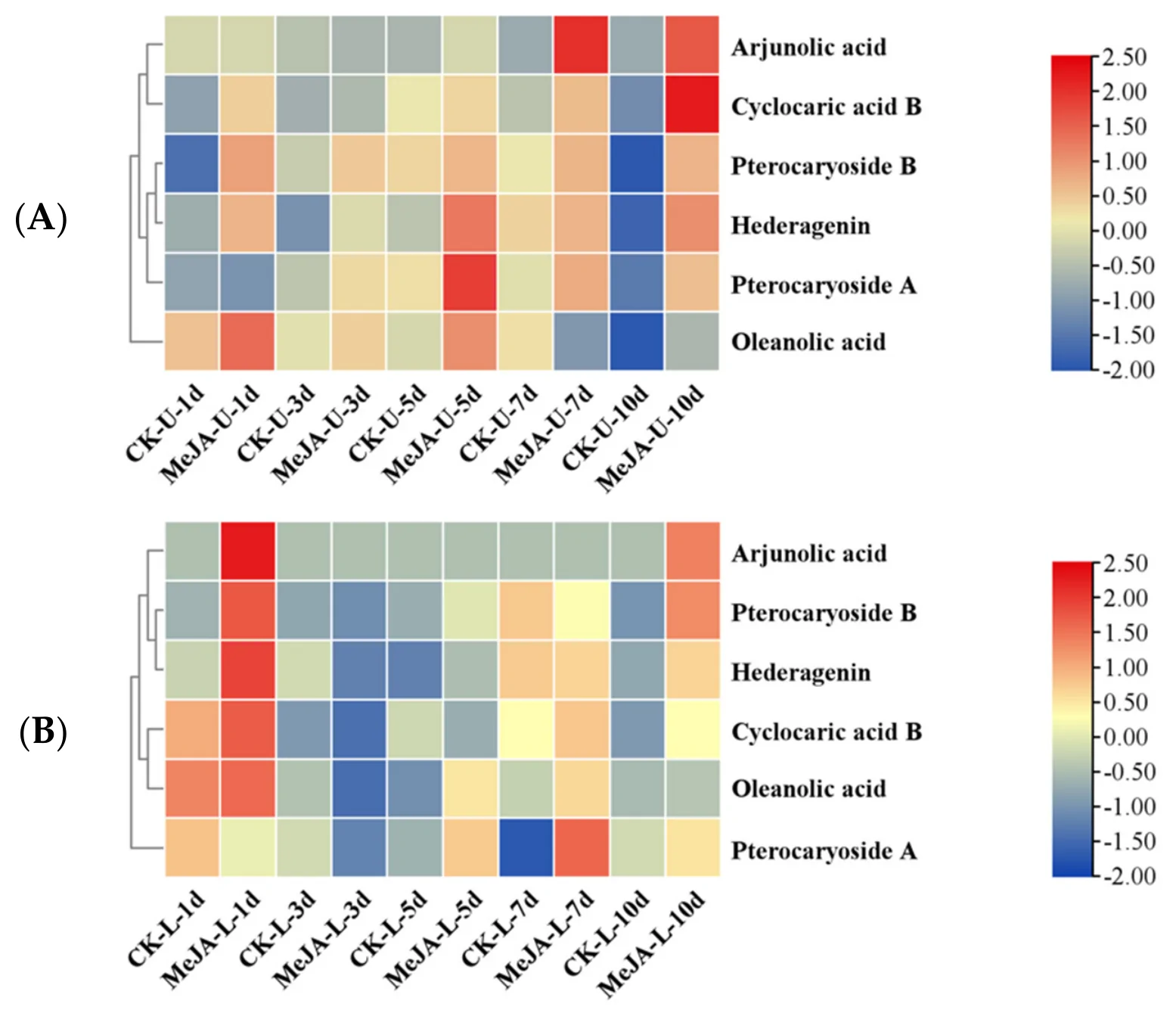
Heatmap showing the effect of MeJA treatment on triterpene monomer content in leaves of *C. paliurus*. (**A**) Effect of MeJA treatment on triterpene monomer content in upper (U) leaves of *C. paliurus*. (**B**) Effect of MeJA treatment on triterpene monomer content in lower (L) leaves of *C. paliurus*.

HPLC analysis demonstrated that MeJA treatment significantly modulated the accumulation of six triterpenoid monomers in *C. paliurus* leaves, exhibiting distinct spatiotemporal patterns between upper and lower leaves (Figure 1). In upper leaves, oleanolic acid and pterocaryoside B levels were markedly elevated after 1 day of treatment. Most monomers continued to increase as treatment progressed, with pterocaryoside A and hederagenin peaking at day 5. By days 7 and 10, a generalized upward trend was observed across most triterpenoids, despite a slight decline in oleanolic acid. Notably, pterocaryoside B and hederagenin concentrations consistently remained significantly higher than the control (CK) throughout the entire experimental period (Figure 1A). Conversely, lower leaves exhibited a more rapid initial response, with most monomers peaking at day 1 (except pterocaryoside A), followed by a transient decline at day 3 and a subsequent resurgence of pterocaryoside A and oleanolic acid at day 5. Although minor fluctuations occurred at days 7 and 10, the concentrations remained overall higher than those in the CK group (Figure 1B). Collectively, MeJA application effectively promoted triterpenoid biosynthesis at 1, 5, and 10 days post-treatment in both leaf positions.

### 2.2. Transcriptome Analysis

#### 2.2.1. Transcriptomic Profiles and Enrichment Analysis in Response to MeJA

**Figure 2:**
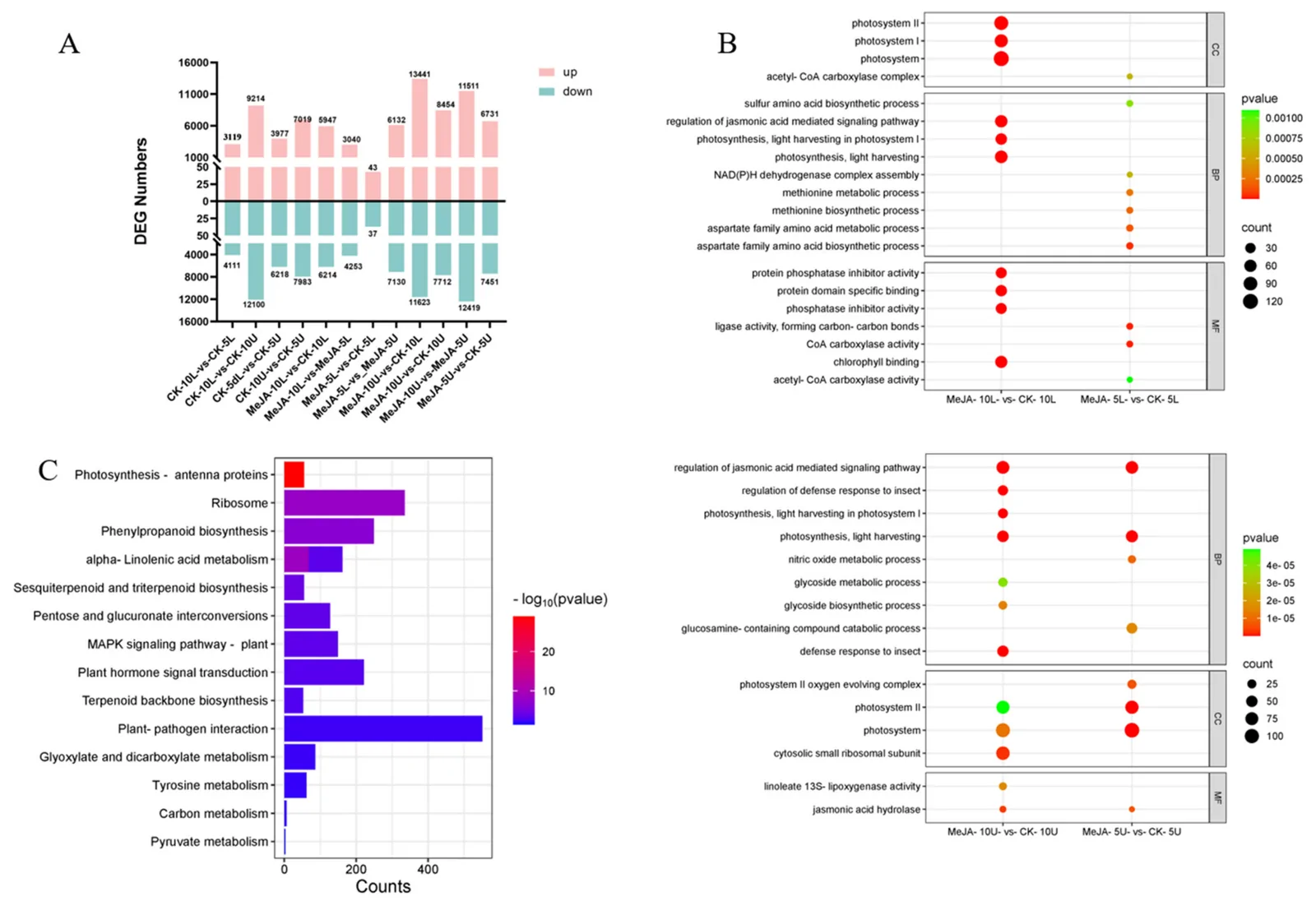
Comprehensive analysis of DEGs. (**A**) The number of upregulated (pink) and downregulated (cyan) DEGs identified in each pairwise comparison group. The x-axis represents different comparison groups, and the y-axis indicates the DEG count. (**B**) Bubble plot for GO enrichment analysis of DEGs. The y-axis shows significantly enriched GO terms, and the x-axis represents comparison groups. The color gradient corresponds to the adjusted *p*-value, and the size of dots indicates the gene count enriched in each GO term. (**C**) Bar plot for KEGG pathway enrichment analysis of DEGs. The y-axis shows significantly enriched KEGG pathways, and the x-axis represents the number of enriched genes. The color of bars represents the −log_10_(*p*-value).

To elucidate the molecular mechanisms underlying MeJA-induced triterpenoid accumulation, transcriptome sequencing was performed on upper (U) and lower (L) leaves at days 5 and 10. Differential expression analysis (Figure 2A) identified substantial transcriptomic reprogramming: the comparison between MeJA-treated and control groups revealed 14,182 and 16,166 DEGs in upper leaves at 5 d and 10 d, respectively. In lower leaves, while only 80 DEGs were detected at 5 d, the number increased sharply to 12,161 by 10 d.

GO enrichment analysis (Figure 2B) categorized these DEGs into biological process (BP), molecular function (MF), and cellular component (CC). In MeJA-treated upper leaves, BP terms were predominantly enriched in “regulation of jasmonic acid-mediated signaling pathway” (GO:2000022) and “photosynthesis, light harvesting” (GO:0009765), while “jasmonic acid hydrolase” (GO:0120091) and “photosystem” (GO:0009521) dominated the MF and CC categories, respectively. Lower leaves exhibited significant enrichment in “photosystem” (GO:0009521) and “aspartate family amino acid biosynthetic process” (GO:0009067).

KEGG pathway analysis (*p* < 0.05) further identified 15 significantly enriched pathways (Figure 2C). Notably, “photosynthesis-antenna proteins” was the most prominent pathway, followed by “ribosome” and “phenylpropanoid biosynthesis.” Crucially, pathways directly related to specialized metabolism, including “terpenoid backbone biosynthesis” and “sesquiterpenoid and triterpenoid biosynthesis,” were significantly enriched. Additionally, the activation of “plant hormone signal transduction” and “MAPK signaling pathways” underscores a complex regulatory network.

#### 2.2.2. Transcriptional Profiling of Triterpenoid Biosynthetic Pathways Under MeJA Induction

Herein, we selected the major expressed transcript of each enzyme based on transcriptome data of *C. paliurus*, in line with earlier studies on triterpenoid biosynthesis of this species. Hence, only these transcripts were visualized in the pathway diagram, and the expression levels of all gene transcripts are summarized in the Supplementary Materials.

(1). Upstream Pathway: Differential Regulation of MVA and MEP Routes

**Figure 3:**
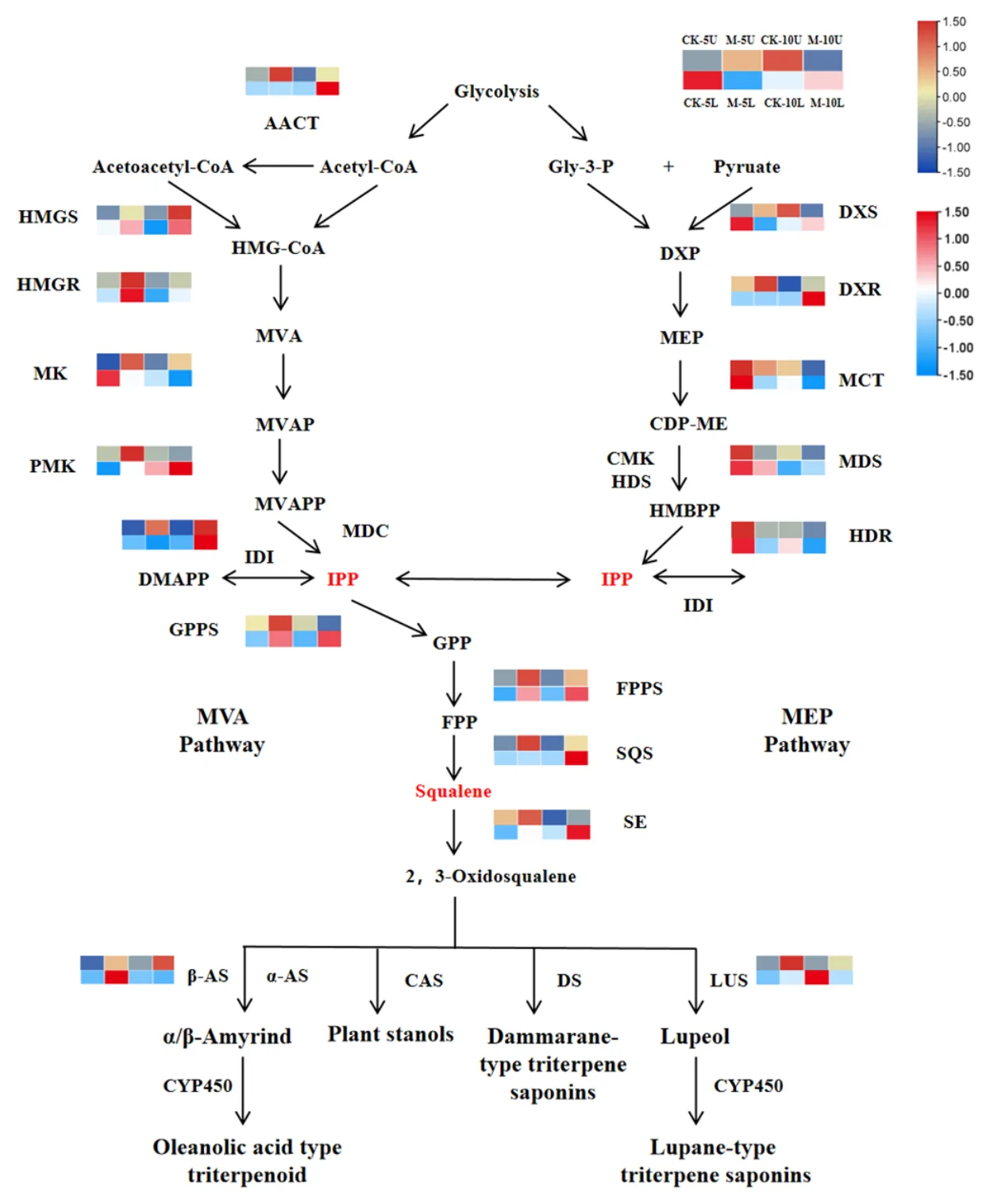
Triterpenoid biosynthetic pathway in *C. paliurus*. The first row of color blocks shows the FPKM values of DEGs from left to right: CK-5U, MeJA-5U, CK-10U, MeJA-10U. The second row displays the FPKM values of differentially expressed genes from left to right: CK-5L, MeJA-5L, CK-10L, MeJA-10L.

To understand the precursor supply for triterpenoid synthesis, we analyzed the expression patterns of genes involved in the MVA and 2-C-methyl-D-erythritol-4-phosphate (MEP) pathways (Figure 3). Following MeJA induction, genes encoding key enzymes in the MVA pathway, including *AACT*, *HMGS*, *HMGR*, and *PMK*, were significantly upregulated, suggesting an enhanced flux through this cytoplasmic route. In contrast, the MEP pathway exhibited a divergent response: while *DXR* expression increased, other core genes such as *DXS*, *MCT*, *MDS*, and *HDR* were suppressed, and *CMK*, *MDC*, and *HDS* maintained basal levels.

As metabolic progression reached the second stage, downstream genes responsible for terpenoid and sterol backbone assembly, including *IDI*, *GPPS*, *FPPS*, *SQS*, and *SE*, exhibited marked transcriptional activation. Interestingly, these genes showed a spatiotemporal shift in peak expression, reaching maximum FPKM values in upper leaves at 5 d post-treatment, whereas peak levels in lower leaves were delayed until 10 d.

(2). Downstream Pathway: Cyclization, Tailoring, and Glycosylation

The downstream diversification of triterpenoids is mediated by oxidosqualene cyclases (OSCs), cytochrome P450s (CYP450s), and UDP-glucosyltransferases (UGTs). Transcriptomic analysis revealed that *β-amyrin synthase* (*β-AS*), a key *OSC* gene, was upregulated in upper leaves at 10 d and in lower leaves at 5 d post-induction. Conversely, *lupeol synthase* (*LUS*) exhibited a downward trend in lower leaves but peaked at 5 d in upper leaves.

Most structural genes involved in skeleton modification (CYP450s) and glycosylation (UGTs) showed differential upregulation, underscoring the broad impact of MeJA on triterpenoid structural tailoring. Notably, three pivotal genes—*CpHMGR*, *CpDXR*, and *CpSQS*—were identified as central regulators. Previous studies and our current FPKM data confirm that *CpSQS*, as the first committed enzyme gene of the downstream pathway, shows a strong positive correlation with total triterpenoid accumulation. Unlike the more ubiquitous upstream genes *CpHMGR* and *CpDXR*, which participate in various primary metabolic processes, *CpSQS* appears to be the primary rate-limiting node for MeJA-induced triterpenoid sequestration in *C. paliurus* [18].

### 2.3. Heterologous Functional Assessment of CpSQS in Tobacco Calli

**Figure 4:**
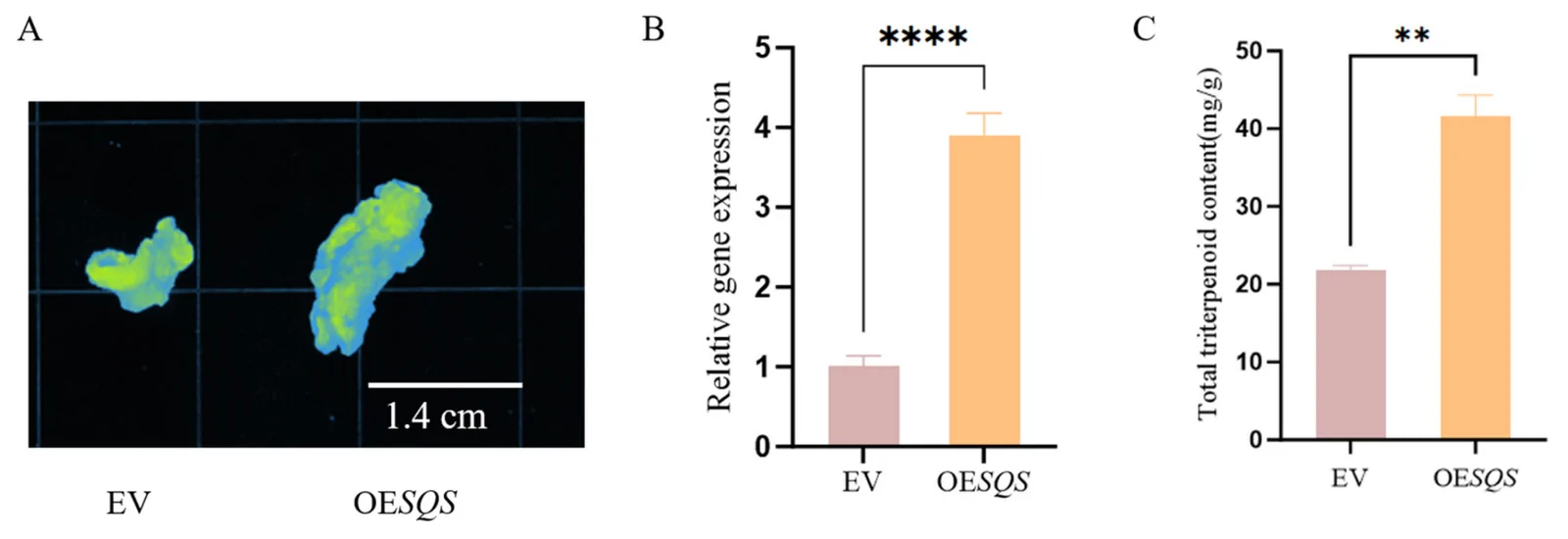
*N. benthamiana* callus fluorescence activity and gene expression. (**A**) In vivo GFP fluorescence imaging of transgenic callus. EV (left) served as the negative control; callus OE*SQS* (right) is shown. Images were acquired with a NEWTON 7.0 BIO FT-500 plant imaging system (Vilber, Collégien, France) in the GFP channel, using an excitation wavelength of 480 nm. The white scale bar represents 1.4 cm. Green–blue pseudocolor indicates GFP fluorescence signal intensity. (**B**) Relative *CpSQS* transcript levels in EV and OE*SQS* callus measured by qRT-PCR. Expression was normalized to *18S rRNA* using the 2^−ΔΔCt^ method. Bars represent the mean of n = 3 biological replicates; error bars indicate SD. (**C**) Total triterpenoid content in EV and OE*SQS* callus. Bars represent the mean of n = 3 biological replicates; error bars indicate SD. Statistics: Pairwise differences between EV and OE*SQS* were assessed by a two-tailed unpaired Student’s *t*-test. ** *p* < 0.01; **** *p* < 0.0001.

Callus tissues were successfully induced from *N. benthamiana* leaf explants via *A. tumefaciens*-mediated transformation (Figure 4). In vivo fluorescence imaging confirmed GFP activity in calli from both the *CpSQS*-overexpressing (OE*SQS*) and empty vector (EV) control groups, validating successful T-DNA integration and reporter gene expression (Figure 4A). qRT-PCR analysis revealed a significant upregulation of *CpSQS* transcript levels in the OE*SQS* calli compared to the EV controls (*p* < 0.0001), confirming effective overexpression of the target gene (Figure 4B). Total triterpenoid content was quantified spectrophotometrically using an oleanolic acid standard curve (y = 0.4141x + 0.0757, R^2^ = 0.9976). The OE*SQS* calli accumulated significantly higher levels of total triterpenoids relative to the EV controls (*p* < 0.01) (Figure 4C). These results demonstrate that heterologous overexpression of *CpSQS* in tobacco calli significantly increased *CpSQS* transcript abundance and total triterpenoid content.

### 2.4. Cloning, in Silico Analysis, and Vector Construction of the CpSQS Promoter

**Figure 5:**
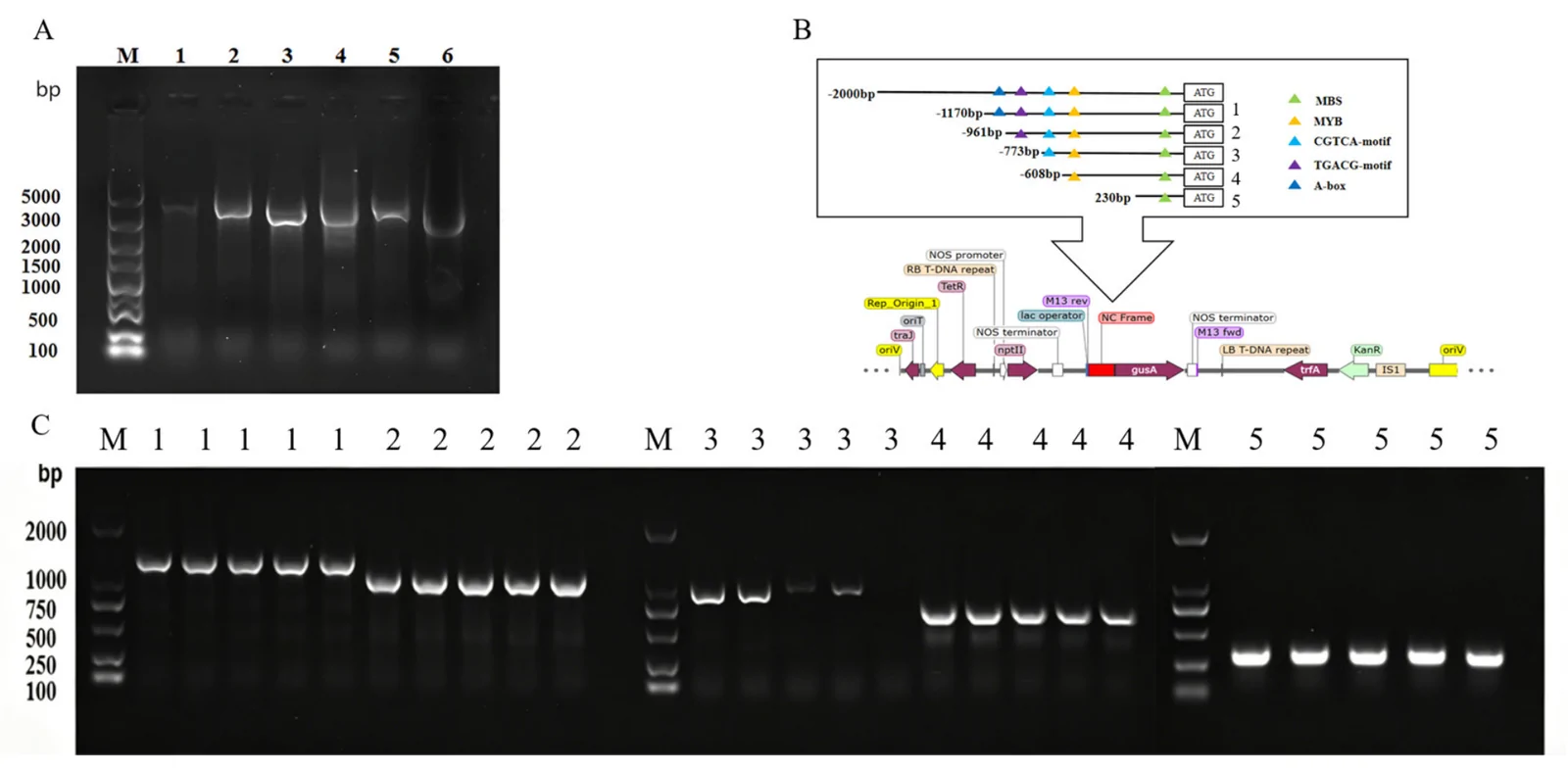
Cloning and vector construction of the *CpSQS* promoter, and positive clone identification. (**A**) PCR amplification of *CpSQS* promoter in *C. paliurus* (M: DL 5000 DNA marker, 1~6: PCR amplicons of the *CpSQS* promoter fragment). (**B**) Schematic diagram of the five recombinant reporter constructs containing serially truncated *CpSQS* promoter fragments fused to the *GUS* gene. Constructs 1~5 correspond to GUS-1170, GUS-961, GUS-773, GUS-608 and GUS-230, respectively. (**C**) Agarose gel electrophoresis for bacterial liquid PCR verification. M: DL 2000 DNA marker; lanes 1~5: PCR products for GUS-1170, GUS-961, GUS-773, GUS-608 and GUS-230, respectively. All agarose gels were stained with Ultra GelRed (10,000×, Vazyme) for DNA visualization.

Using genomic DNA of the *C. paliurus* template, PCR amplifications were performed with three forward and two reverse primers, respectively, yielding six expected bands of approximately 3 kb (Figure 5A). The most distinct 3161 bp band was recovered, cloned into the pCE3 blunt-end vector, and transformed into *Escherichia coli* DH5α. Colony PCR and subsequent plasmid sequencing confirmed that the cloned sequence was identical to the predicted promoter sequence.

Bioinformatic analysis using the PlantCARE and PLACE databases predicted a total of 39 cis-acting elements in the *CpSQS* promoter region (Table S5). In addition to core promoter elements such as the TATA box, the promoter harbors light-responsive elements (e.g., G-box), hormone-responsive motifs (including ABRE, CGTCA-motif, and P-box), and an anaerobic-response element (ARE). The analysis also revealed multiple tissue-specific regulatory motifs (e.g., seed-, leaf-, and root-associated motifs) as well as putative binding sites for transcription factor families such as WRKY, bHLH, and MYB.

Five 5′-deletion promoter fragments were designed: QF1 (−1170 to −1 bp) retains the A-box, TGACG-motif, CGTCA-motif, MYB, and MBS; QF2 (−961 to −1 bp) lacks the A-box; QF3 (−773 to −1 bp) further lacks the TGACG-motif; QF4 (−608 to −1 bp) contains only the MYB and MBS; and QF5 (−230 to −1 bp) retains only the MBS. PCR amplification using the promoter-containing plasmid as template, followed by electrophoresis and sequencing, confirmed that the sizes and sequences of all fragments matched the expected design. Subsequently, each fragment was inserted upstream of the GUS reporter gene in a plant expression vector via seamless cloning, generating a series of GUS–promoter fusion constructs (Figure 5B). After transformation into *E. coli* DH5α, colony PCR and sequencing verified the correct insertion and sequence integrity of all constructs. The resulting plasmids, designated GUS-1170, GUS-961, GUS-773, GUS-608, and GUS-230, were successfully prepared for subsequent promoter-activity assays. Finally, the recombinant plasmids were introduced into *A. tumefaciens* competent cells using the freeze–thaw method. Positive clones were selected and validated by colony PCR. Electrophoretic analysis confirmed that all constructs were successfully transformed into *A. tumefaciens* (Figure 5C), completing the preparatory steps for subsequent plant transformation and promoter-activity analysis.

### 2.5. Histochemical Staining for GUS Activity and Quantitative Analysis of GUS Activity in N. benthamiana

**Figure 6:**
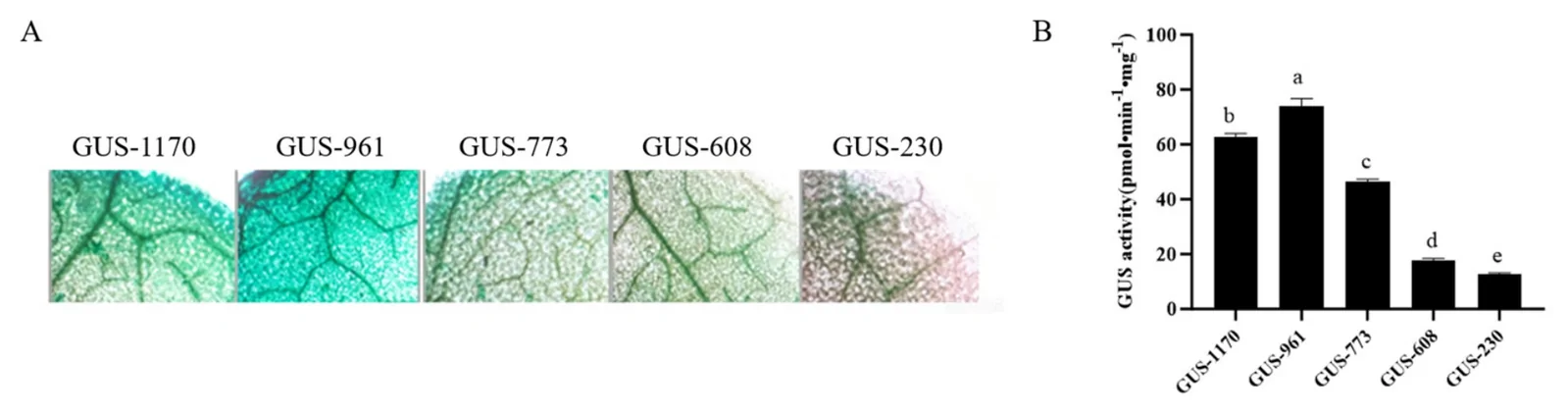
Histochemical staining and quantitative analysis of GUS activity driven by different promoter deletion fragments in *N. benthamiana*. (**A**) GUS histochemical staining of leaf disks transiently transformed with the five truncated promoter constructs (GUS-1170, GUS-961, GUS-773, GUS-608 and GUS-230, from left to right). GUS histochemical staining was performed using a GUS staining kit (Coolaber, Beijing, China). (**B**) Quantitative analysis of GUS activity for each promoter deletion construct. Bars represent the mean of n = 3 biological replicates; error bars indicate SD. One-way analysis of variance (ANOVA) followed by Duncan’s multiple range test was used for statistical analysis. Different lowercase letters indicate significant differences at *p* < 0.05.

Histochemical GUS staining revealed that all five promoter deletion fragments (GUS-1170, GUS-961, GUS-773, GUS-608, and GUS-230) drove visible GUS expression in infiltrated *N. benthamiana* leaves, indicating basal promoter activity in each construct (Figure 6A). Quantitative fluorometric GUS assays further demonstrated consistent trends with the staining results (Figure 6B).

## 3. Discussion

### 3.1. Spatiotemporal Regulation of Triterpenoid Biosynthesis in C. paliurus by MeJA

MeJA, a key signaling molecule in plant secondary metabolism, has been widely documented to modulate terpenoid biosynthesis across diverse plant species. The present study shows that MeJA treatment significantly upregulates genes in the MVA pathway *(CpAACT*, *CpHMGS*, *CpSQS*, etc.) in *C. paliurus,* and promotes the accumulation of both individual triterpene monomers (e.g., oleanolic acid, ursolic acid) and total triterpenes. Notably, immature upper leaves and mature lower leaves exhibited distinct spatiotemporal responses to MeJA: upper leaves showed peak responses at 5 days post-treatment, while lower leaves peaked as early as 1 day, indicating differential metabolic regulation between leaves at different developmental stages. Collectively, MeJA treatment represents an effective strategy for augmenting specific triterpenoid monomers in *C. paliurus*.

In *Taraxacum antungense*, MeJA activates the bHLH transcription factor *TaMYC2*, which directly binds to the promoters of key triterpene-synthesis enzyme genes, thereby promoting their transcription and triterpene precursor synthesis [22]. In *Panax ginseng*, MeJA induces the NAC transcription factor *PgNAC72*, positively regulating the biosynthesis of ginsenosides [23]. In *Platycodon grandiflorus*, MeJA, through the root-specific transcription factor *PgbHLH28,* activates the downstream MVA/MEP dual-pathway rate-limiting enzyme genes *PgHMGR2* and *PgDXS2*, substantially increasing triterpenoid saponin accumulation in roots [24]. In *Rosa Roxburghii*, low-concentration MeJA significantly increases ursane-type triterpenoid content by activating key genes (e.g., *HMGR*, *SQS*, *AS*) in the root triterpene synthesis pathway. It also induces a “hyper-hairy root” phenotype to coordinate growth and defense functions. Triterpenoid accumulation in roots directly regulates development, whereas in stems and leaves it primarily mediates stress responses [25]. These findings indicate that MeJA-mediated induction of terpenoid synthesis is evolutionarily conserved, yet the responsive patterns and target genes are often tissue-specific.

Transcriptomic analysis further revealed that MeJA treatment triggered a large number of differentially expressed genes in *C. paliurus* leaves. The transcriptional response was more pronounced in upper leaves (14,182 DEGs at 5 days; 16,166 DEGs at 10 days) compared with lower leaves, which responded with a delay (80 DEGs at 5 days; 12,161 DEGs at 10 days). GO enrichment analysis indicated that genes involved in the “jasmonic acid-mediated signaling pathway” and “photosynthesis” were significantly enriched in upper leaves, whereas lower leaves were enriched for “photosystem” and amino acid biosynthetic processes. The observed enrichment of photosynthesis- and primary-metabolism-related GO terms further suggests that MeJA triggers broad adaptive physiological responses beyond secondary metabolism, implying divergent physiological adaptation strategies in different leaf positions: upper leaves may prioritize activation of the JA signaling pathway to enhance secondary metabolism, while lower leaves focus on maintaining photosynthetic and primary metabolic homeostasis [18]. KEGG pathway enrichment further confirmed that terpenoid backbone biosynthesis, sesquiterpene/triterpene synthesis, and plant hormone signal transduction pathways were significantly activated, providing pathway-level evidence for MeJA-promoted triterpenoid accumulation.

With respect to the biosynthetic routes, MeJA primarily activated the cytoplasmic MVA pathway (with upregulation of *CpAACT*, *CpHMGS*, *CpHMGR* and *CpPMK*) and the downstream backbone-assembly genes (*CpIDI*, *CpFPPS*, *CpSQS* and *CpSE*), whereas the plastidial MEP pathway showed a divergent response with only *CpDXR* induced. This indicates that MeJA mainly bolsters triterpenoid precursor supply in *C. paliurus* through the MVA route. Together, these transcriptomic results provide a molecular framework for understanding the MeJA-mediated regulation of triterpenoid biosynthesis in *C. paliurus*.

### 3.2. Functional Characterization of CpSQS in Triterpenoid Biosynthesis

Triterpenoid biosynthetic genes typically exist as multi-gene families in plant genomes, and individual members may exhibit marked tissue-specific differences in expression and functional contribution; often only one predominantly expressed paralog makes a major contribution to pathway flux [26]. Consistent with this notion, our genome reanalysis identified one full-length SQS locus per haplotype in the diploid accession QQL006, whereas at least three and four full-length SQS-like loci were recovered from the current phased assemblies of the tetraploid accessions Cpa_TCP_BNU2022 and tetraPA, respectively. Despite this copy-number complexity, repeated amplification using primers designed within conserved SQS regions consistently yielded the same full-length transcript, *CpSQS*, which was also among the predominantly expressed SQS transcripts in our transcriptome dataset. Combined with the strong positive correlation between *CpSQS* expression and total triterpenoid accumulation observed in the present and previous studies [18], these observations provided the rationale for prioritizing *CpSQS* for functional characterization. Unlike the more ubiquitous upstream MVA-pathway genes *CpHMGR* and *CpDXR*, which participate in various primary metabolic processes, *CpSQS* appears to be a key rate-limiting node for MeJA-induced triterpenoid accumulation in *C. paliurus*.

SQS acts as the first committed enzyme in the triterpenoid and sterol biosynthetic pathways, catalyzing the condensation of two molecules of farnesyl diphosphate (FPP) to form squalene. Its critical role in modulating secondary metabolite accumulation has been established across multiple plant species [8]. In the present study, heterologous overexpression of *CpSQS* in *N. benthamiana* calli led to a significant increase in total triterpenoid content, directly confirming its regulatory function in promoting triterpenoid biosynthesis. This finding aligns with functional reports of SQS orthologs in other species. For example, in alfalfa, MeJA-induced expression of *MsSQS* and its overexpression enhanced total saponin accumulation, demonstrating its essential role in triterpenoid saponin production [27]. Similarly, heterologous expression of *Arabidopsis AtSQS* in *Centella asiatica* trichomes elevated squalene and centelloside levels while reducing phytosterol content, indicating a metabolic flux shift toward triterpenoid synthesis [28]. In *Withania somnifera*, overexpression of *WsSQS* promoted withanolide biosynthesis, whereas its silencing led to reduced accumulation of squalene, phytosterols, and withanolides, underscoring its central role in pathway flux regulation [29].

Although tobacco calli provided a rapid and controllable system for preliminary functional assessment, they do not fully reproduce the developmental and tissue-specific metabolic regulation of intact *C. paliurus* plants. Furthermore, the present findings do not establish *CpSQS* as the only functional or rate-limiting SQS member, and the possible contributions of other low-abundance or sequence-divergent SQS copies require further investigation. Additional validation in regenerated transgenic plants will be required once an efficient transformation and regeneration system becomes available.

### 3.3. Functional Dissection of the CpSQS Promoter Reveals a Repression–Enhancement Combinatorial Regulatory Pattern

Promoters, located upstream of the 5′ end of genes, regulate transcriptional initiation by recruiting the transcriptional machinery and integrating regulatory signals, thereby contributing to the timing, magnitude, and context specificity of gene expression [30]. The composition and spatial organization of cis-regulatory elements are therefore important determinants of tissue-specific, developmental, and environmentally responsive transcription. In the present study, a 2000 bp sequence upstream of the *CpSQS* coding region was cloned and subjected to functional analysis. In silico prediction identified multiple hormone-, light-, and tissue-responsive cis-elements, including canonical MeJA-responsive TGACG and CGTCA motifs, together with putative binding sites for WRKY, MYB, and bHLH transcription factors. These features suggest that *CpSQS* transcription may integrate multiple endogenous and environmental signals. In particular, the presence of WRKY-binding motifs is consistent with findings in *Camellia oleifera*, where *CoWRKY15* binds to the *CoSQS* promoter and positively regulates squalene biosynthesis [31], supporting a potential role of WRKY-mediated regulation in SQS transcription.

Functional dissection using a series of 5′-deletion constructs further revealed distinct regulatory properties within the *CpSQS* promoter. All truncated fragments retained detectable GUS activity, indicating that basal transcriptional capacity was maintained in the shorter promoter regions. However, deletion of the −961 to −773 bp region caused a marked reduction in GUS activity, indicating that this 188 bp segment contributes substantially to high-level promoter activity and may contain positive regulatory elements or binding sites for transcriptional activators. In silico analysis identified putative MYB-binding sites within this region, including MYBCORE motifs. MYB transcription factors have been implicated in the regulation of triterpenoid biosynthesis in tea and grapevine [32,33], raising the possibility that MYB-family proteins participate in the activation of *CpSQS* through this promoter region. Nevertheless, direct binding of specific MYB proteins to this sequence remains to be experimentally verified.

Conversely, the GUS-961 construct displayed higher reporter activity than the longer GUS-1170 construct, suggesting that the −1170 to −961 bp interval may contain negative regulatory elements. Taken together, the deletion analysis supports a putative repression–enhancement combinatorial pattern within the *CpSQS* promoter, in which an upstream region between −1170 and −961 bp may restrain transcriptional activity, whereas the adjacent −961 to −773 bp region contributes positively to promoter activation. Such an arrangement could provide a mechanism for fine-tuning *CpSQS* expression in response to developmental status, leaf position, and MeJA treatment. However, because these regulatory properties were inferred from promoter deletion assays, the identities of the corresponding transcription factors and the direct functions of individual cis-elements require further validation.

Importantly, the regulation of triterpenoid biosynthesis is unlikely to depend solely on cis-regulatory elements within individual promoters. In the present study, several genes associated with the mevalonate and triterpenoid biosynthetic pathways, including *HMGR*, *FPPS*, *SQS*, and *SE*, exhibited coordinated transcriptional responses to MeJA, suggesting that pathway-level regulation may involve additional mechanisms beyond gene-specific promoter control. Functional clustering of metabolically related genes has been documented across eukaryotic systems [34] and increasing evidence indicates that genes involved in plant specialized metabolism can also occur as physically linked biosynthetic gene clusters [35]. Such genomic organization may provide a structural basis for coordinated regulation of multiple pathway genes. Moreover, the transcriptional behavior of plant metabolic gene clusters is closely associated with chromatin state. Yu et al. [36] demonstrated that plant metabolic gene clusters can be delineated by characteristic chromatin signatures, including H3K27me3 and the histone variant H2A.Z, which are associated with transcriptional repression and activation, respectively. Consistent with the broader importance of chromatin-level regulation, coordinated transcription of functional gene clusters in yeast has also been shown to depend on multiple chromatin remodelers [37].

These findings provide a broader regulatory framework for interpreting the coordinated MeJA-responsive transcription observed in *C. paliurus*. Our promoter deletion assays demonstrate that local cis-regulatory regions contribute substantially to *CpSQS* transcription, particularly the positive regulatory region between −961 and −773 bp and the putative negative regulatory region between −1170 and −961 bp. At the same time, the coordinated expression of multiple triterpenoid-related genes raises the possibility that triterpenoid biosynthesis may also be influenced by higher-order regulatory mechanisms involving genomic organization and chromatin dynamics. However, the present study does not establish whether these genes are physically clustered in the *C. paliurus* genome or whether their MeJA-responsive transcription is accompanied by coordinated changes in chromatin state. Therefore, the proposed involvement of gene clustering or chromatin-level regulation should be regarded as a working hypothesis rather than a demonstrated mechanism. Future integration of chromosome-level genomic information with chromatin accessibility, histone modification, and transcription factor–DNA interaction analyses will be necessary to determine how promoter-specific and higher-order regulatory mechanisms collectively shape the spatial and temporal regulation of triterpenoid biosynthesis in response to MeJA.

## 4. Materials and Methods

### 4.1. Plant Materials

Four-year-old *C. paliurus* seedlings were cultivated at Wuhan Polytechnic University (30.621° N, 114.268° E). Leaves were classified according to phenological stage and growth position into upper immature leaves and lower mature leaves [19]. The leaves were treated by spraying with a 0.2 mmol·L^−1^ MeJA solution. Leaves were collected at 1, 3, 5, 7, and 10 days post-treatment. A portion of the samples was dried for triterpenoid content determination, while another portion was rapidly frozen in liquid nitrogen and stored at −80 °C for RNA extraction and transcriptome sequencing. In addition, 4-week-old *N. benthamiana* plants were subjected to the same 0.2 mmol·L^−1^ MeJA treatment for transient expression analysis.

### 4.2. Determination of Six Triterpenoid Monomers

The content of six major triterpene monomers (arjunolic acid, cyclocaric acid B, pterocaryoside B, pterocaryoside A, hederagenin, and oleanolic acid) in leaves of *C. paliurus* was determined by high-performance liquid chromatography (HPLC), following a previously described method [38]. Analyses were performed on a Waters e2695 Alliance HPLC system coupled with a Waters 2489 UV detector and Empower 3 software, using an X-Bridge C18 column (250 × 4.6 mm, 5 μm, Waters). Chromatographic separation was performed using a mobile phase composed of 0.01% (*v*/*v*) formic acid in water (solvent A) and 0.01% (*v*/*v*) formic acid in acetonitrile (solvent B). The gradient elution program was set as follows: 0–13 min, 8–19% B; 13–28 min, 19–21% B; 28–42 min, 21–50% B; and 42–46 min, 50% B. The flow rate was 1.0 mL/min, the column temperature was maintained at 45 °C, and detection was performed at 205 nm.

### 4.3. Transcriptome Sequencing and Analysis

RNA extraction was performed using the E.Z.N.A.^®^ Plant RNA Kit (Omega Bio-tek, Norcross, GA, USA). Subsequently, the concentration and purity of the extracted RNA were testedby Denovix DS-11 FX+ spectrophotometer (DeNovix, Inc., Wilmington, DE, USA). The extracted RNA was used for subsequent experiments.

Qualified RNA samples were used for library construction. The resulting libraries were subjected to paired-end sequencing on an Illumina high-throughput sequencing platform (MetWare Biotechnology Co., Wuhan, China). The high-quality reads obtained from sequencing were aligned with the assembled transcripts, and the fragments per kilo base of transcript per million mapped reads (FPKM) was calculated to evaluate gene expression. Differentially expressed genes (DEGs) were identified using the criteria of |log_2_(fold change)| ≥ 1 and a false discovery rate (FDR) ≤ 0.05. Gene Ontology (GO) functional enrichment analysis and KEGG pathway enrichment analysis were performed (*p*-value < 0.05).

### 4.4. CpSQS Cloning and Overexpression Vectors Construction

#### 4.4.1. Cloning of *CpSQS*

First-strand cDNA was synthesized from total RNA using HiScript II Q RT SuperMix for qPCR (+gDNA wiper) (Vazyme Bio, Nanjing, China). Following validation via 1% agarose gel electrophoresis, high-fidelity polymerase chain reaction (PCR) amplification was performed using PrimeSTAR HS DNA Polymerase (TaKaRa, Inc., Dalian, China) with the *C. paliurus* cDNA as a template. PCR amplification was performed under the following conditions: denaturation at 98 °C for 10 s, annealing at 55 °C for 15 s, and extension at 72 °C for 1 min 30 s per cycle. The reaction was run for 30 cycles, followed by a final hold at 4 °C. Specific primers for the *CpSQS* coding sequence were designed based on transcriptome data (Table S1).

#### 4.4.2. Overexpression of *CpSQS*

Using the sequencing-verified correct clone as a template, the *CpSQS* CDS fragment was amplified with primers featuring universal adapters (Uni forward *SQS*/Uni reverse *SQS*) (Table S1). Employing the Nimble Cloning kit (NC Bio, Nanjing, China), the target fragment was seamlessly cloned into the plant overexpression vector pNC-Cam2304-MCS35S to construct the recombinant vector *CpSQS*-pNC-Cam2304 [39]. A control empty vector pNC-Cam2304 was simultaneously constructed. The recombinant plasmid was validated for correctness via colony PCR and sequencing.

### 4.5. N. benthamiana Genetic Transformation and Total Triterpenoid Analysis

#### 4.5.1. Genetic Transformation

The constructed *CpSQS* overexpression vector and the pNC-Cam2304 empty vector (as a control) were transformed into *Agrobacterium tumefaciens* GV3101 competent cells, respectively. The resulting bacterial culture was then used to transform sterile *N. benthamiana* seedlings via the leaf disk method to induce callus formation. After 40 days, the *N. benthamiana* callus was harvested, immediately flash-frozen in liquid nitrogen, and subsequently freeze-dried. The lyophilized samples were divided into two portions: one for triterpenoid content determination and the other for quantitative real-time PCR (qRT-PCR) analysis.

#### 4.5.2. Determination of the Total Triterpenoid Content

The total triterpenoid content in the freeze-dried callus of *N. benthamiana* was determined using a colorimetric method [40]. A reference system was established using an oleanolic acid standard solution. Briefly, the callus extract was evaporated at 60 °C. Subsequently, vanillin-glacial acetic acid and perchloric acid were added for color development at 60 °C for 15 min. After adjusting the volume with ethyl acetate, the absorbance was measured at 550 nm. Using oleanolic acid as the standard, the calibration curve was generated as y = 0.4141x + 0.0757 (R^2^ > 0.99).

#### 4.5.3. Gene Expression Analysis by qRT-PCR

qRT-PCR assays were performed using a LineGene 9600 Plus PCR System (Bioer Technology Co., Ltd., Hangzhou, China). The 20 µL reaction mixture comprised the following: 10 µL Taq Pro Universal SYBR qPCR Master Mix, 2 µL cDNA, 0.4 µL each of forward and reverse primers, and 7.2 µL nuclease-free water. All reactions were carried out in triplicate, and *18S rRNA* was used as the endogenous control for normalization. The thermal profile included an initial denaturation step (95 °C for 30 s), 40 cycles of amplification (95 °C for 10 s, 60 °C for 15 s), and a final melting curve stage (95 °C for 15 s, 60 °C for 1 min, 95 °C for 15 s) to verify primer specificity. Gene expression was quantified via the comparative 2^−ΔΔCt^ method. Primer sequences are listed in Table S2.

### 4.6. Cloning and Analysis of the CpSQS Promoter

#### 4.6.1. Promoter Cloning and Bioinformatics Analysis

Genomic DNA of *C. paliurus* was isolated from leaves using the CTAB method. To ensure precise amplification of the target gene’s promoter, the reverse primer (Table S3) was designed based on a 200 bp sequence downstream of the putative start codon (ATG). The promoter fragments were amplified using PrimeSTAR GXL DNA polymerase (TaKaRa, Inc., Dalian, China), and the PCR products were purified using FastPure Gel DNA Extraction Mini Kit (Vazyme Bio, Nanjing, China). The purified DNA was then subcloned into pCE3 Blunt Vector (Vazyme Bio, Nanjing, China), and plasmids were obtained using the FastPure Plasmid Mini Kit (Vazyme Bio, Nanjing, China). The plasmids were subsequently sequenced by Sangon Biotech Co., Ltd. (Shanghai, China). The complete promoter sequence was assembled using SeqMan software Pro v7.1.0 (DNASTAR, Madison, WI, USA). The cloned promoter sequence was analyzed for the prediction of cis-acting elements using the PlantCARE (http://bioinformatics.psb.ugent.be/webtools/plantcare/html/, accessed on 1 January 2026) database.

#### 4.6.2. Construction of 5′-End Deletion *CpSQS* Promoter Fragments

Based on the predicted cis-acting element distribution, five pairs of specific primers (QF1-QF5/DP) were designed to amplify 5′-end deletion fragments of varying lengths: QF1 (−1170bp to −1bp), QF2 (−961 bp to −1 bp), QF3 (−773 bp to −1 bp), QF4 (−608 bp to −1 bp), and QF5 (−230 bp to −1 bp) (Table S4). PCR amplification was performed using 2× Phanta Flash Master Mix (Vazyme Bio, Nanjing, China) with a plasmid containing the full-length promoter as template. Using the Nimble Cloning kit, each deletion fragment was separately cloned upstream of the GUS reporter gene in the pNC-Cam2304-MCS35S vector to construct promoter–GUS fusion vectors, designated: GUS-1170, GUS-961, GUS-773, GUS-608, and GUS-230. Recombinant plasmids were validated through colony PCR and sequencing. The successfully constructed overexpression vector (*CpSQS*-pNC-Cam2304, empty vector pNC-Cam2304) and each promoter deletion fusion vector (GUS-1170 to GUS-230) were separately transformed into *A. tumefaciens* GV3101 competent cells using the freeze–thaw method. Following transformation, plates containing YEP medium supplemented with Kanamycin Sulfate (Solarbio, Beijing, China) (50 mg/L) and Rifampicin (Yuanye Bio-Technology, Co., Ltd., Shanghai, China) (25 mg/L) were spread. Plates were incubated at 28 °C for 48–72 h. Single colonies were selected for colony PCR validation.

### 4.7. Transient Transformation in N. benthamiana and Β-Glucuronidase (Gus) Activity Analysis

#### 4.7.1. Transient Expression

The recombinant vectors were introduced into *A. tumefaciens* GV3101. The bacterial suspension was infiltrated into the abaxial side of leaves from 4-to-6-week-old *N. benthamiana* plants [41]. Three to four leaves per plant were infiltrated. Following infiltration, plants were subjected to dark incubation for 24 h, then transferred to standard photoperiodic conditions (16 h light/8 h dark) for 48 h.

#### 4.7.2. Gus Histochemical Staining Analysis

GUS histochemical staining of *N. benthamiana* leaves was performed using a GUS staining kit (Coolaber, Beijing, China). Samples infiltrated with the GUS gene lacking the CaMV35S promoter served as the negative control. The 50× GUS staining solution was diluted to 1× working concentration with staining buffer. Leaf disks (6 mm in diameter) were excised from *N. benthamiana* leaves, immersed in the 1× staining solution, and subjected to vacuum infiltration. Staining was carried out by incubation at 37 °C in the dark overnight. Following staining, samples were destained with a gradient of 75% ethanol until the background pigment of the negative control was completely removed. GUS activity was indicated by the presence of blue precipitates against a white tissue background.

#### 4.7.3. Quantitative Gus Activity Assay in *N. benthamiana*

(a) Total protein extraction and quantification

Approximately 100 mg of fresh leaf tissue was pulverized in liquid nitrogen and homogenized in 1 mL of protein extraction buffer. The homogenate was centrifuged, and the supernatant was collected as the crude protein extract. Protein concentration was determined using the Bradford method [42]. A standard curve was generated using BSA standards (0–500 μg/mL). Aliquots of 20 μL of standards or samples were mixed with 200 μL of diluted Coomassie Brilliant Blue G-250 dye in a 96-well plate. After 5 min incubation at room temperature, absorbance was measured at 595 nm.

(b) GUS enzymatic activity measurement

A standard curve was prepared using 4-methylumbelliferone (4-MU) diluted in stop solution (1–10 μM) [43]. Fluorescence was measured at ex/em 365/455 nm. For the enzyme assay, 150 μL of protein extract was mixed with 150 μL of 1 mM 4-MUG substrate and incubated at 37 °C. Aliquots of 100 μL were taken at 10, 20, and 30 min and transferred to 900 μL of stop solution, and fluorescence was immediately measured. GUS activity was calculated as nmol 4-MU produced per min per mg of protein (pmol·min^−1^·mg^−1^) based on the 4-MU standard curve and the linear phase of the reaction.

## 5. Conclusions

This study elucidates the functional role of *CpSQS* and delineates the core regulatory region of its promoter, providing a significant foundation for uncovering the molecular mechanisms underlying triterpenoid saponin biosynthesis in *C. paliurus*. Through integrated transcriptomic, functional, and promoter analyses, we systematically investigated the effects of exogenous MeJA on triterpenoid synthesis and accumulation in *C. paliurus* leaves. MeJA treatment enhanced total triterpenoid content, promoted triterpenoid saponin biosynthesis, and exerted spatiotemporally differential effects on triterpenoid monomer accumulation, with upper leaves exhibiting a particularly pronounced response.

Transcriptome analysis demonstrated that key genes of the MVA pathway—including *CpAACT*, *CpHMGS*, *CpHMGR*, and *CpSQS*—were upregulated following MeJA treatment, indicating that MeJA enhances triterpenoid production primarily through induction of the MVA pathway. Functional validation via heterologous overexpression in *N. benthamiana* confirmed that *CpSQS* positively regulates triterpenoid accumulation. Moreover, *CpSQS* expression correlated significantly with multiple transcription factors from the MYB and WRKY families, and in silico analysis of its promoter identified jasmonate-responsive elements as well as binding sites for MYB and WRKY factors. Deletion-based promoter assays further pinpointed the −961 bp to −773 bp region as essential for maintaining high transcriptional activity, revealing a “repression–enhancement” combinatorial architecture that likely fine-tunes *CpSQS* expression in response to developmental and environmental signals.

Collectively, these results establish *CpSQS* as a regulatory node in the triterpenoid biosynthetic pathway of *C. paliurus* and uncover a spatially organized promoter structure that governs its precise expression. This work not only advances the understanding of triterpenoid regulation in this medicinal species, but also provides a molecular framework for future efforts to enhance triterpenoid production through genetic or elicitor-based strategies.

Nevertheless, some limitations exist in the current research. The biological function of *CpSQS* was validated by heterologous expression in *N. benthamiana*, whereas the in vitro enzymatic activity assay of recombinant *CpSQS* protein was not performed. Subsequent in vitro catalytic characterization will help further reveal the biochemical property of *CpSQS*.

## Data Availability

Raw RNA-Seq data from this study are deposited in the NCBI (National Center for Biotechnology Information) SRA repository, accession number PRJNA1471466 (https://dataview.ncbi.nlm.nih.gov/object/PRJNA1471466, accessed on 28 May 2026). Data will be made available on request.

## Supplementary Materials

The following supporting information can be downloaded at https://www.mdpi.com/article/10.3390/ijms27188432/s1.
