# Supplementary material for: Functional Characterization and Promoter Analysis of a MeJA-Responsive Squalene Synthase Gene CpSQS from Cyclocarya paliurus (Batal.) Iljinsk
Source: Int J Mol Sci. 2026 Sep 21;27(18):8432. doi: 10.3390/ijms27188432 (PMC13607441; doi:10.3390/ijms27188432)
Supplement: Supplementary file 1 [file ijms-27-08432-s001.zip › ijms-4525854-supplementary corrected/Highlights.pdf]

Highlights

1 *CpSQS* was verified as a key candidate gene for triterpenoid synthesis.

2 Methyl jasmonate induces expression of the *CpSQS* gene.

3 Identify a 180+ bp core enhancer region in the *CpSQS* promoter.
