# Supplementary material for: Functional Characterization and Promoter Analysis of a MeJA-Responsive Squalene Synthase Gene CpSQS from Cyclocarya paliurus (Batal.) Iljinsk
Source: Int J Mol Sci. 2026 Sep 21;27(18):8432. doi: 10.3390/ijms27188432 (PMC13607441; doi:10.3390/ijms27188432)
Supplement: Supplementary file 1 [file ijms-27-08432-s001.zip › ijms-4525854-supplementary corrected/Supplementary Material.pdf]

Supplementary materials

Functional characterization and promoter analysis of a MeJA-responsive squalene synthase gene *CpSQS* from *Cyclocarya paliurus* (Batal.) Iljinsk.

Juan Chen[a, †],Qinghui Xia[a, †],Yulan Dong [a],Yuhang Pan [a], Quanwei Lv [a],Jinbo Huang [a] ,Shaopeng Zhang[a,b],Xingxing Dong[a,b, *],Xiaoling Chen[a,b,*]

[a]School of Modern Industry for Selenium Science and Engineering, Wuhan Polytechnic University, Wuhan 430023, China

[b]National R&D Center for Se-Rich Agricultural Products Processing Technology, Wuhan Polytechnic University, Wuhan 430023, China

[†] These authors contributed equally to this work.

[*]Correspondence: dongxingxinghg@whpu.edu.cn;chenxl0811@whpu.edu.cn.

Table S1. Primers used for cloning and overexpression of *CpSQS*.

Primers	Sequence (5′→3′)
SQS-F	ATGGGGAGCTTGGGTGCGGT
SQS-R	TCAATTATTTGGTCGTTTGGCAGAG
Uni_forward_*SQS*	AGTGGTCTCTGTCCAGTCCTATGGGGAGCTTGGGTGC
Uni_reverse_*SQS*	GGTCTCAGCAGACCACAAGTTCAAAATCATTGCTCAATCC
*M13*F	CGCCAGGGTTTTCCCAGTCACGAC
*M13*R	AGCGGATAACAATTTCACACAGGA

Table S2. Primers for the quantitative real-time PCR (qRT-PCR).

Primers	Sequence (5′→3′)
CpSQS F	GAACAGGCTGGATGCGATAC
CpSQS R	TCAATTATTTGGTCGTTTGG
18S F	AGTATGGTCGCAAGGCTGAAA
18S R	CAGACAAATCGCTCCACCAA

Table S3. Primers applied in *CpSQS* promoter cloning experiment.

Primers	Sequence (5′→3′)
UP1	TGTGGCTACGACTTGTGCTCTAGG
UP2	ATGCGCTCGTACTCTATCCTACTT
UP3	TCCTTGTTTGTTGGCTTTGACTTTCTT TCAT
DP1	CTTGTGGAGCATGGTGTAGCAGAAA GCC
DP2	AGGGATCTGCTTCTCAGCATGCCT
M13 F	CGCCAGGGTTTTCCCAGTCACGAC

Note: UP: Upstream Primer; DP: Downstream Primer. Primers UP1-UP3 and DP1-DP3 were used for full-length promoter cloning, while M13 F was applied for PCR validation of bacterial culture.

Table S4. Primers for amplifying 5′ -end deletion fragments of the *CpSQS* promoter.

Primers	Sequence (5′→3′)	Objective
QF1	AGTGGTCTCTGTCCAGTCCTTCTTACTAGTTG GTTCGACT	Clone of the missing fragment QF1
QF2	AGTGGTCTCTGTCCAGTCCTTGACCATTACCC ACATTGA	Clone of the missing fragment

		QF2
QF3	AGTGGTCTCTGTCCAGTCCTTCCTGTCGACCTC CATACGCAACCT	Clone of the missing fragment QF3
QF4	AGTGGTCTCTGTCCAGTCCTTCGTCGTGAATGG ACAGCAG	Clone of the missing fragment QF4
QF5	AGTGGTCTCTGTCCAGTCCTTAACGTACGG ATCAGACTTTGCA	Clone of the missing fragment QF5
DP	GGTCTCAGCAGACCACAAGTTCTTTTTCGT TAAATCGGATTTCG	Cloning of missing fragments

Table S5. Partial cis-elements of *CpSQS* promoter predicted by PlantCARE and PLACE

Cis-acting element	Sequence	Function
TATA-Box	ATATAA/ATATAT/ATTATA /TATA/TATATA/TATAAA	Transcription starts at −30bp to initiate the promoter core element
CAAT-Box	CAAT/CAAAT/CCAAT	Common acting elements in the promoter and enhancer regions
ARE	AAACCA	Anaerobically inducible element
TATC-box	TATCCCA	Gibberellin response element
TGA-element	AACGAC	Auxin response element
TGACG-motif	TGACG/TGACG/TGACG	MeJA response element
CGTCA-motif	CGTCA	MeJA response element
ABRE	ACGTG	Abscisic acid response element
ERE	ATTTTAAA	Ethylene response element
MBS	CAACTG	*MYB*-binding site
G-Box	CACGTT/CACGTC/CACGAC	Light-responsive element
EBOXBNNAPA	CANNTG	*bHLH* binding site;seed specific
MYCCONSENSUSAT	CANNTG	*bHLH* binding site;leaf specific

MYBST1	GGATA	*MYB* binding site
MYBPZM	CCWACC	*MYB* binding site
MYB1AT	WAACCA	*MYB* binding site
MYBCORE	CNGTTR	*MYB* binding site;flavonoid biosynthesis,drought stress
MYBCOREATCYCB1	AACGG	*MYB* binding site
WBOXHVISO1	TGACT	*WRKY* binding site
WRKY710S	TGAC	*WRKY* binding site
WBOXATNPR1	TTGAC	*WRKY* binding site;stress responsive cis element
WBBOXPCWRKY1	TTTGACY	*WRKY* binding site
